# Development of biodegradable Zn-Mn-Li and CaP coatings on Zn-Mn-Li alloys and cytocompatibility evaluation for bone graft

**DOI:** 10.3389/fbioe.2022.1013097

**Published:** 2022-09-14

**Authors:** Hui-Fen Qiang, Zhao-Yong Lv, Cai-Yao Hou, Xin Luo, Jun Li, Kun Liu, Chun-Xiu Meng, Wan-Qing Du, Yu-Jue Zhang, Xi-Meng Chen, Feng-Zhen Liu

**Affiliations:** ^1^ Department of Materials Science and Engineering, Liaocheng University, Liaocheng, China; ^2^ Liaocheng People’s Hospital, Dongchangfu Hospital of Liaocheng Hospital, Liaocheng, China

**Keywords:** zn-based alloys, mechanical property, degradation property, cytocompatibility, osteogenesis

## Abstract

Zn-based alloys are considered as new kind of potential biodegradable implanted biomaterials recently. The difficulty of metal implanted biomaterials and bone tissue integration seriously affects the applications of metal implanted scaffolds in bone tissue-related fields. Herein, we self-designed Zn0.8Mn and Zn0.8Mn0.1Li alloys and CaP coated Zn0.8Mn and Zn0.8Mn0.1Li alloys, then evaluated the degradation property and cytocompatibility. The results demonstrated that the Zn0.8Mn0.1Li alloys had profoundly modified the degradation property and cytocompatibility, but Zn0.8Mn0.1Li alloys had particularly adverse effects on the surface morphology of osteoblasts. The results furtherly showed that the CaP-coated Zn0.8Mn and Zn0.8Mn0.1Li alloys scaffold had better biocompatibility, which would further guarantee the biosafety of this new kind of biodegradable Zn-based alloys implants for future clinical applications.

## 1 Introduction

In recent years, biodegradable ferroalloy and magnesium alloy as the main representatives of medical metal materials with biodegradable properties have attracted extensive attention (Gu et al., 2012). The unique surface and mechanical properties of metals make them more attractive implanted scaffolds. At present, magnesium alloy and ferroalloy are widely studied, but they all have certain clinical defects, which limit their applications in the clinical biodegradable field (Li et al., 2021).

Zn-based biodegradable materials have been introduced in the last few years. Zinc is a common nutrient in the human body and plays a momentous role in immunity, the nervous system, and so on (Yang et al., 2022). The response of host tissue is the most critical factor in determining the success of biodegradable bone implantation, and the response of host tissue is largely determined by its degradation behavior. Based on the chemical activity of element, Zn is between Mg and Fe (the standard electrode potential of Mg is −0.237V, Fe is −0.440V, and Zn is −0.763 V), it can be preliminarily inferred that the degradation rate of Zn is slower than Mg and faster than Fe, which meets the requirements of being degradable metal biomaterials from corrosiveness. Therefore, the research on the design and biocompatibility evaluations of Zn-based alloys will have great significance. In terms of orthopedic implants, pure Zn has low hardness and cannot meet the requirements of clinical implants (Zhang et al., 2021). However, the addition of alloying elements can effectively solve this problem (Yin et al., 2019). Alloying elements such as Mn, Li, Cr, Sn, and Ge that exhibit eutectic reactions at the Zn-rich end are preferred to avoid large brittle intermetallic compounds (Shi et al., 2020; Zhang et al., 2019). Li is an important alloying element, it can refine the grain size of Zn-based alloys and improve the mechanical properties of Zn-based alloys, and the addition of Li can reduce the mass of pure Zn because the density of Li is the lowest among alloying elements (Zhao et al., 2017). Li group reported the microstructure evolution, mechanical properties, *in vitro* degradation, and cytocompatibility evaluations of ZnLi alloys, which had well optimal scaffold performance (Li et al., 2020). Sun group reported that this new Zn-Mn-Mg alloy had the mechanical and biodegradable properties required for ligament reconstruction fixation (Sun et al., 2021). But until now, Zn-Mn-Li alloys were not reported.

Various chemical, physical and biological cues have been revealed to regulate the adhesion and differentiation of stem cells (Yao et al., 2013a; Yao et al., 2021). Surface properties of biomaterials such as roughness, surface morphology, and element composition can influence ambient cells (Yao et al., 2013b). Surface properties can be modified with micro-arc oxidation, ion implantation, bionic deposition, chemical transformation, and so on (Jablonská et al., 2016). Osseointegration is a complex process. Bai group reported that changing the surface properties of biodegradable implant materials, such as morphology and composition, can modulate their osteogenic properties and accelerate bone healing (Bai et al., 2021). Phosphate chemical conversion technology is a process of chemical and electrochemical combination, which is used to form CaP coatings and bind them firmly to substrates (Zhao et al., 2021a; Zhao et al., 2021b). In the process of coatings design, elements with bone promoting function can be introduced, and the coatings have high crystallinity and stability, and the degradation rate and the ion release are slow, which guarantees the long-term stability of the implants. In addition, the degradation product PO_4_^3−^ is also a major component of bone and can participate in bone mineralization (Zhao et al., 2019). The CaP coatings composited immediately on the Zn implants surface can adjust the corrosion resistance, roughness, and hydrophilicity of Zn-based alloys, create a suitable environment for cell survival and effectively improve the adhesion of osteoblasts (Su et al., 2019; Acheson et al., 2022). Calcium is used in a variety of biomaterials and is a major ingredient in the body’s bones (Ling et al., 2021). Moreover, degradation products of calcium-rich phosphate not only contribute to the integration of Zn implants with host bone, but also regulate the microenvironment of tissues around implants and promote new bone formation (Wang et al., 2021). Theoretically, the surface modification of Zn-based alloys by phosphate chemical conversion technology has a broad application prospect.

Therefore, in this study, alloying elements manganese and lithium will be added to the zinc matrix, and the mechanical properties, degradation behavior, and cytocompatibility of Zn, Zn0.8Mn, and Zn0.8Mn0.1Li alloys will be compared longitude, simultaneously, CaP coatings on the surface of Zn-based alloys were synthesized by phosphate chemical conversion technology. The degradation and osteogenic ability of CaP coatings were studied. This study will further optimize the biosafety of novel biodegradable Zn-based alloys implants and provide a theoretical basis for future clinical applications.

## 2 Materials and methods

### 2.1 Materials and coating processes

**FIGURE 1:**
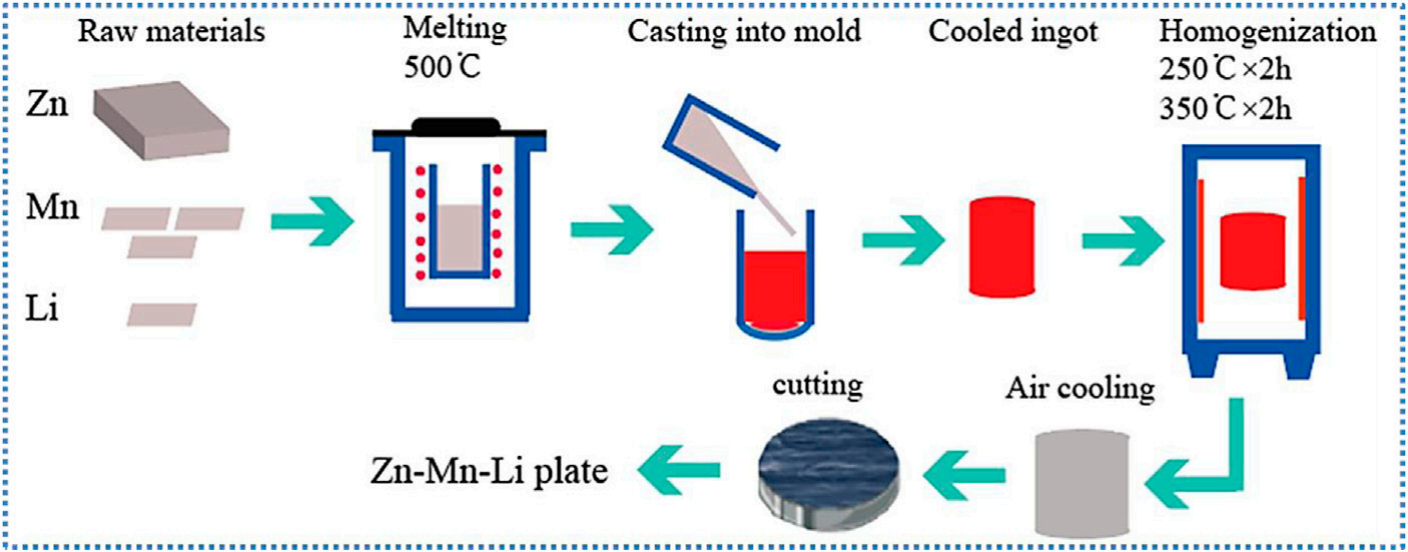
Schematic diagram of the alloys preparation route.

Zn0.8Mn, Zn0.8Mn0.1Li alloys were prepared from high purity Zn, Mn, and Li metal powders of 99.95 wt%. Figure 1 briefly showed the preparation process of the alloys. Under the protection of the Ar atmosphere, put the raw materials into a ZG−0.01 vacuum induction furnace, kept the constant temperature at 500°C for 40 min, placed it at room temperature, and then poured it into the graphite mold to obtain the cylindrical ingot. All cylindrical ingots were homogenized at 250°C for 2 h, then at 350°C for 2 h, and finally put into the furnace for cooling. The actual compositions of the alloy were measured by ICP-AES (Thermo Science M Series). The results were shown in Table 1.

**TABLE 1:** Actual composition of Zn-based alloys.

Types of alloy	Zn/wt%	Mn/wt%	Li/wt%
Pure Zn	99.99	—	—
Zn0.8Mn	Balance	0.82	—
Zn0.8Mn0.1Li	Balance	0.82	0.11

**FIGURE 2:**
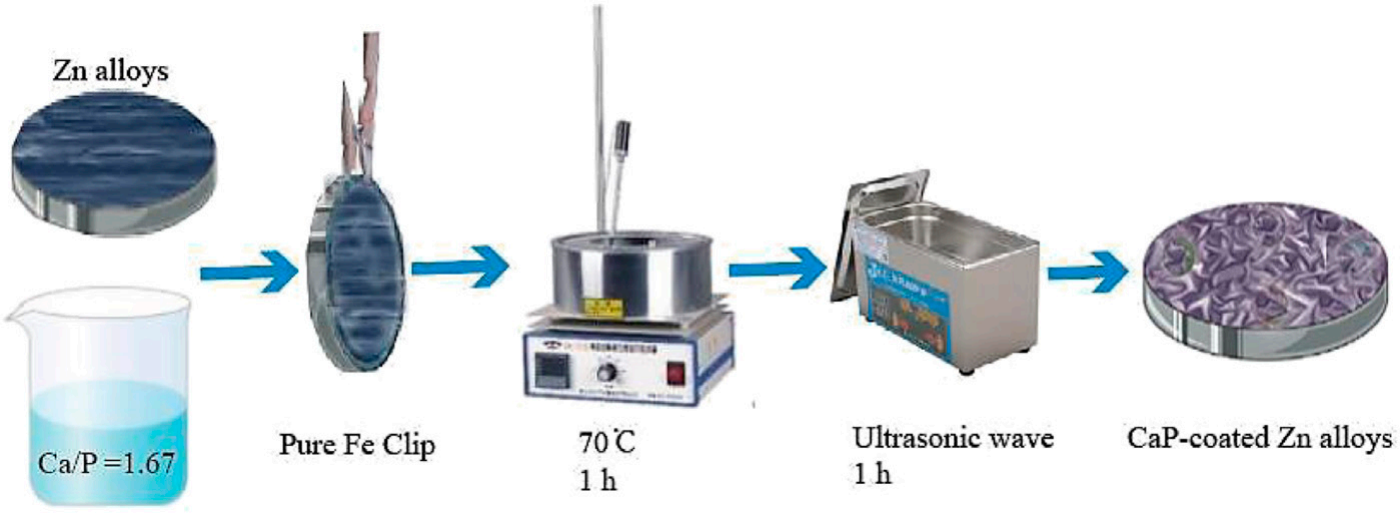
Flow diagram of the experimental procedure.

The pure Zn, Zn0.8Mn alloy, and Zn0.8Mn0.1Li alloy were cut into a disk with a diameter of 10 mm and a thickness of 1 mm, then grounded with 240–5,000 silicon carbide sandpaper, and polished to obtain a smooth surface. Ultrasonic cleaning was then performed in acetone, alcohol, and deionized water. As Figure 2 shown, the prepared Zn disk was connected to the pure Fe clip and then acid etched for pretreatment. The pretreated samples were then immersed in a phosphate chemical conversion solution with the design composition, in which the ratio of calcium to phosphorus was 1.67 at 70°C for 60 min. The pH of the phosphate chemical conversion solution was adjusted to 2.5–3.5 using H_3_PO_4_ or Ca(OH)_2_. Then, placed the reaction beaker in an ultrasonic cleaner for 60 min. In the last, the samples were cleaned with deionized water and dried at room temperature.

### 2.2 Characterizations of Zn-based alloys and CaP coatings on Zn-based alloys

The phase composition of the samples was measured by XRD (Rigaku D-max/2500 PC, Japan). The surface morphology of all samples was watched by FE-SEM (SU-4800, Hitachi, Japan), and the microstructure was observed by FE-SEM after etching with 4% nitric acid solution. Under the condition of 300 g load and 10 s loading time, the microhardness of the materials was measured by microhardness tester. For the accuracy of the results, we have tested at least five parallel samples. The wetting angle was measured with a contact angle meter to evaluate the surface wettability of the sample (DSA100S, KRUSS, Germany).

XRD was employed to detect the crystal structure of the coatings. The surface morphology of the CaP coatings was manifested using FE-SEM. The surface chemical constituent was determined using the FE-SEM equipped with EDS. The surface wettability was evaluated by DSA100S, and the surface roughness was analyzed by atomic force microscope (AFM, spa-300hv).

### 2.3 *In Vitro* degradation test

The electrochemical properties of the materials were tested by an electrochemical analyzer (modular XM, AMETEK, United States). Working electrode, placed on a specific mold, with an exposed study surface area of 0.237 cm^2^. Before measurement, the samples were soaked in SBF solution for 30 min to obtain a stable surface state. The open circuit potential was set to 1,800 s to achieve stability. The potential dynamic polarization (PDP) test was performed in the range of ±0.3 V. Constant scanning rate was 1 mVs^−1^. Electrochemical impedance spectroscopy (EIS) was performed in the frequency range of 10^5^ Hz to 10^–2^ Hz. ZSimpWin software was used to analyze the impedance data and fit it with the equivalent curve.

Early corrosion behaviors of Zn, Zn0.8Mn, and Zn0.8Mn0.1Li were researched in SBF at 37 ± 0.5°C for 14 and 28 days. Microscopical images and chemical compositions were obtained using FE-SEM equipped with EDS. The corrosion products were analyzed by XRD with radiation and 4°/min scanning speed.

### 2.4 *In Vitro* biosafety test

#### 2.4.1 The sample processing

Before the tests, the samples were polished and soaked in 75% ethanol solution for 20 min, followed by UV irradiation for 20 min.

#### 2.4.2 Mouse embryonic osteoblast precursor cells culture

The osteoblast precursor cell line (MC3T3-E1) was selected for cell tests. The culture medium was α-DMEM (Gibco, United States) supplemented with 10% FBS (Gibco, United States), 1% L-glutamine, and 10% penicillin-streptomycin at 37°C in 5% CO_2_ atmosphere.

#### 2.4.3 Cytotoxicity test

After the cells were cultured on the scaffold for 1, 3, and 7 days, 10% CCK-8 reagent was added and placed in an incubator with 5% CO_2_ at 37°C for 3 h. The cellular activity was assessed on a microtiter plate reader (Thermo, America).

#### 2.4.4 Cell adhesion test

The 2 × 10^5^ MC3T3-E1 cells were inoculated for 3 days and fixed in 4% paraformaldehyde (Sangon Biotech) for 30 min. Washed three times and then dehydrated with 30%, 50%, 75%, 90%, 95%, and 100% gradient ethanol (Biogenic organism) solutions for 5 min each time. Finally, it dried slowly at room temperature. The sample morphology was observed by FE-SEM. And cell adhesion numbers were performed with DAPI (Sigma) after cultured for 3 days.

#### 2.4.5 Cell viability test

The 2 × 10^5^ MC3T3-E1 cells were inoculated for 1, 3, and 7 days. Added 2 µl of Calcein-AM Solution (2 mM) and 6 µl of Propidium iodide (PI, 1.5 mM) to 1×buffer and mixed well. Then added 200 µl mixture to the surface of the Zn-based alloys and stained it for 20 min without light. Finally viewed under a fluorescence microscope (Nikon, Japan).

#### 2.4.6 Cell morphology evaluation

The 2 × 10^5^ MC3T3-E1 cells were inoculated for 1, 3, and 7 days. Then, the cells were fixed with 4% paraformaldehyde for 15 min and infiltrated with 0.1% Triton for 20 min. Finally, 200 μl podophyllotoxin dye and 100 μl 1% bovine serum albumin were dyed at room temperature for 30 min.

#### 2.4.7 Measurement of Alkaline Phosphatase Activity and extracellular matrix mineralization

Osteogenic differentiation was induced by adding osteogenic induction solution (β-glycerophosphate, ascorbic acid, and dexamethasone) into the complete medium. After 7 days of culture, alkaline phosphatase (ALP) activity was detected by BCIP/NBT ALP Color Development Kit (Beyotime), and after 14 days of culture, extracellular matrix mineralization was detected by alizarin red S(Sigma) staining.

#### 2.4.8 Cytokine determinations of MC3T3-E1

To further investigate the effects of different scaffold materials on the osteogenic expression of MC3T3E1, the expression of RUNX2 and COL-1 were detected by real-time quantitative polymerase chain reaction (RT-qPCR) after the cells were cultured on the scaffold for 7 days. Total RNA was extracted by TRIzol reagent (Life Technologies). Complementary DNA was synthesized from 1,000 ng of total RNA using the SensiFAST™ cDNA Synthesis Kit (Byolin, Australia) according to the manufacturer’s protocol. The gene expression was then quantified using an SYBR Green quantitative polymerase chain reaction kit (Invitrogen) in a 7500 RT-PCR system (QuantStudio™, applied biosystems) with a procedure of 95°C for 3 min and 95°C for 3 s, followed by 40 cycles at 60°C for 30 s. Gene expression was normalized to GAPDH using the ΔΔCt method. The primer sequences were shown in Table 2.

**TABLE 2:** Primer sequences used in the study.

Gene	Primer	Sequences (5′-3′)
RUNX2	Forward	AAA​TGC​CTC​CGC​TGT​TAT​GAA
	Reverse	GCTCCGGCCCACAAATCT
COL-1	Forward	GCTGGAGTTTCCGTGCCT
	Reverse	GACCTCGGGGACCCATTG
GAPDH	Forward	TGA​CCA​CAG​TCC​ATG​CCA​TC
	Reverse	GAC​GGA​CAC​ATT​GGG​GGT​AG

### 2.5 Statistical analysis

Using SPSS 22.0 statistical software, The data were demonstrated as means ± standard deviation (SD). Using one-way ANOVA to analyze the statistical difference. The distinction was significant at *p* < 0.05.

## 3 Results

### 3.1 Characterizations of Zn-based alloys

**FIGURE 3:**
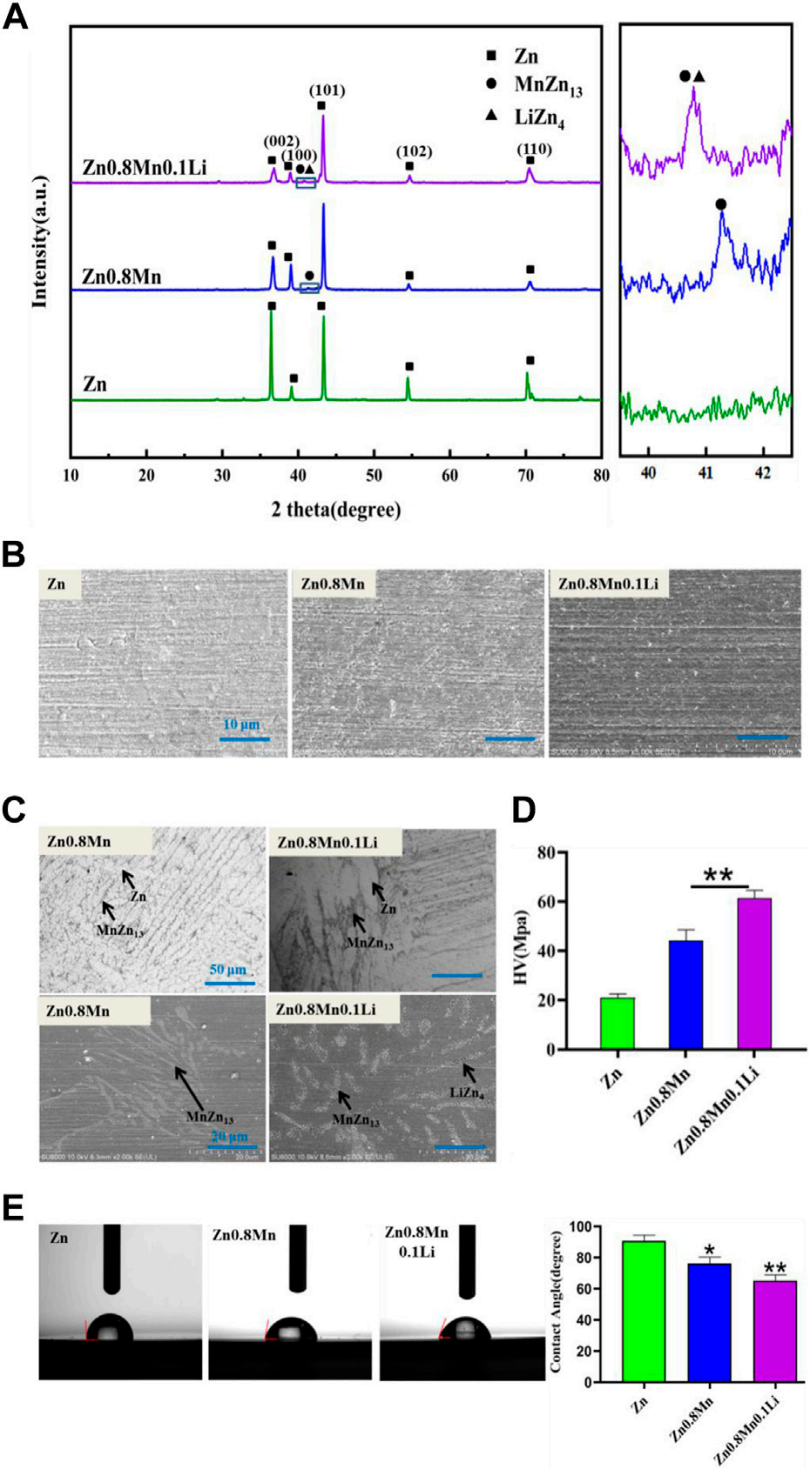
Characterizations of Zn, Zn0.8Mn, and Zn0.8Mn0.1Li. **(A)** X-ray diffraction patterns. **(B)** Surface morphologies by FESEM. **(C)** The metallographic structure. **(D)** Microhardness. **(E)** Water contact angles.*p < 0.05 compared to pure Zn, **p < 0.01 compared to pure Zn.

Pure Zn, Zn0.8Mn, and Zn0.8Mn0.1Li alloys had been selected for phase analysis and biological analysis *in vitro*. Figure 3A showed the XRD patterns of Zn-based alloys. MnZn_13_ phase was detected in Zn0.8Mn, and MnZn_13_ and LiZn_4_ phases were detected in Zn0.8Mn0.1Li alloys. Figure 3B presented the surface morphologies of Zn-based alloys. As can be seen, Zn, Zn0.8Mn, and Zn0.8Mn0.1Li had smooth surface morphology. As we can see from Figure 3C, MnZn_13_ and LiZn_4_ were detected in the Zn0.8Mn0.1Li alloy. LiZn_4_ particles attached to MnZn_13_ particles and formed MnZn_13_/LiZn_4_ compound structure. By comparing the two figures, the addition of Li refined the microstructure significantly. Systematically, the microhardness of the alloy was significantly increased from 20.94 HV of pure Zn to 61.2 HV of Zn0.8Mn0.1Li alloy (Figure 3D). Figure 3E showed a digital image of the droplet morphology and the matching calculation of the contact angle. The contact angle of Zn, Zn 0.8Mn, and Zn0.8Mn0.1Li decreased in order. They were 90.6 ± 4.32°, 76.4 ± 4.47°, and 65.3 ± 4.1°, respectively. Mainly because after adding alloying elements, the eutectic structure was formed and the grain was refined, the contact angle was reduced and wettability was improved.

### 3.2 Degradation of Zn-based alloys

**FIGURE 4:**
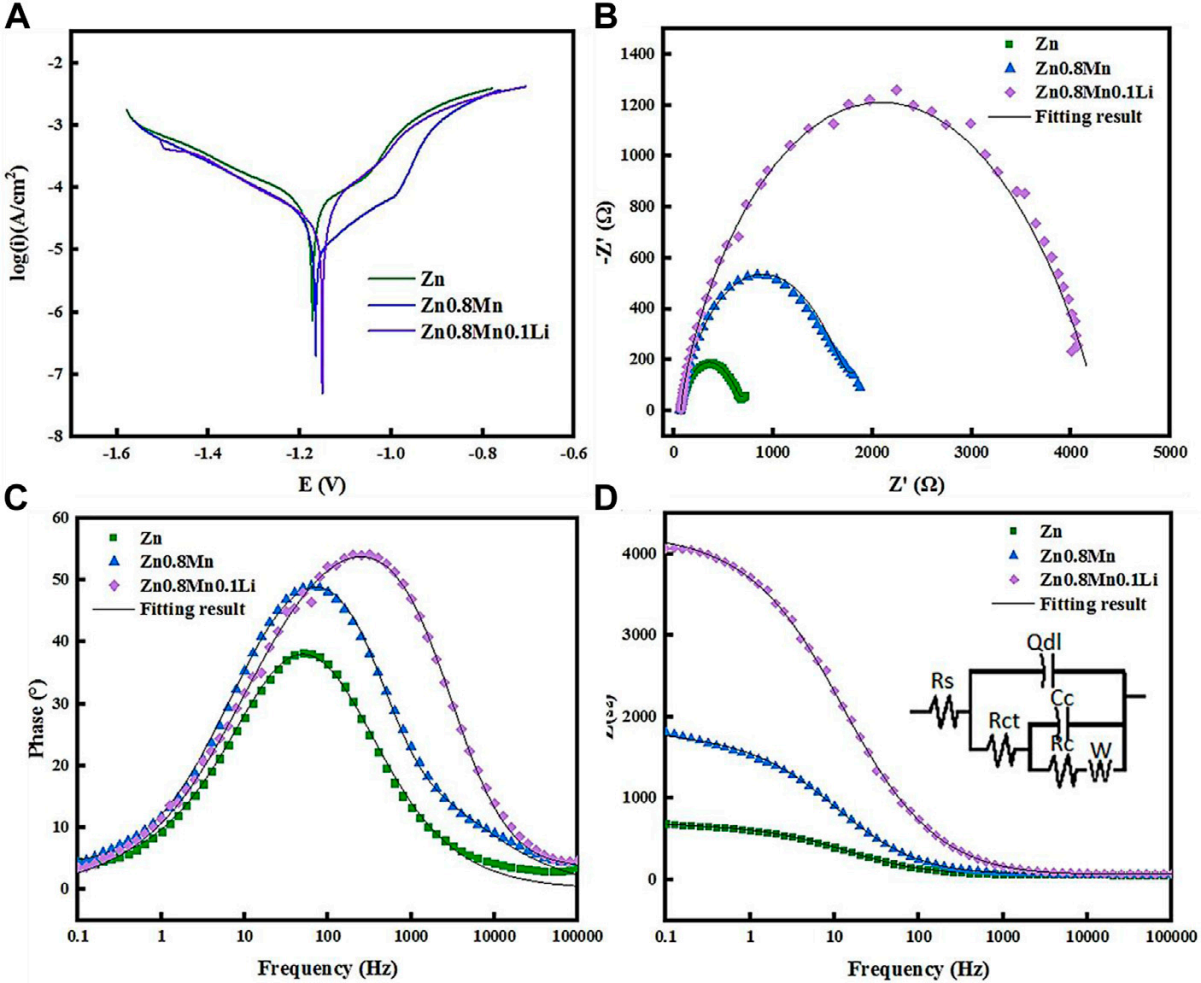
Polarization curves and EIS results of pure Zn and Zn-based alloys in SBF. **(A)** Potentiodynamic polarization curves. **(B)** Nyquist plots. **(C)** Bode plots of phase angle vs. Frequency. **(D)** Bode plots of |Z| vs. Frequency. The inset in Figure 4D is an equivalent circuit for fitting the EIS spectra.

The polarization curves of pure Zn, Zn0.8Mn, and Zn0.8Mn0.1Li were given in Figure 4A. The fitting results were shown in Table 3. Conduct PDP test to compare the corrosion resistance of samples. The slopes of anode and cathode Tafel curves changed with the change of Mn/Li content, which indicated that Mn/Li affected the dissolution and oxygen consumption of Zn matrix. Among all materials, the Ecorr value of Zn was the lowest (−1.17 VCE). As for Zn0.8Mn and Zn0.8Mn0.1Li, Ecorr values increased with Mn/Li content. And the icorr values of Zn0.8Mn were lower than that of Zn0.8Mn0.1Li. The corrosion rate (CR) of Zn0.8Mn0.1Li was 0.046 mm/y, which was slower than that of pure Zn. Figure 4B showed the EIS plots of pure Zn, Zn0.8Mn, and Zn08Mn0.1Li. As shown in the Nyquist curves (Figure 4B), compared to pure Zn, Zn0.8Mn0.1Li had a bigger semicircle diameter, which indicated its lower corrosion rate. After adding Mn/Li, Zn0.8Mn0.1Li showed the characteristics of diffusion impedance in the low-frequency scope, the slope of the curve in the low-frequency scope increased, and the smallest frequency impedance modulus (|z|) increased (Figures 4C,D). The fitted equivalent circuit was shown in Figure 4D. Rs, Rct, and Rc were solution resistance, charge transferring resistance between samples and SBF solution, and resistance of corrosion products layer, respectively. Qc represented the capacitance of the corrosion products layer. Qdl was a constant phase element (CPE) corresponding to the double layer capacitance. There were many reasons for CPE, such as non-uniformity, electrode pores, surface roughness, slow adsorption reaction, uneven distribution of potential and current, et al. The fitting results were shown in Table 4. The R_C_ of the alloy can be calculated as Zn0.8Mn0.1Li > Zn0.8Mn > Zn descending sort, so as their corrosion resistances.

**TABLE 3:** Polarization data of pure zinc and zinc alloy samples in SBF.

Materials	βa (mV/dec)	−βc (mV/dec)	Ecorr (V/SCE)	Icorr (μA/cm2)	CR (mm/a)
Zn	166.30 ± 21.57	288.7 ± 0.55	−1.1710	53.70	0.085
Zn0.8Mn	175.55 ± 3.51	265.36 ± 0.58	−1.1565	30.90	0.055
Zn0.8Mn0.1Li	125.96 ± 0.83	248.14 ± 0.95	−1.1504	26.92	0.046
CaP Zn	64.83 ± 1.04	253.57 ± 6.42	−0.9532	3.45	0.00059
CaP Zn0.8Mn	124.88 ± 1.55	272.63 ± 1.15	−0.9394	7.74	0.0014
CaP Zn0.8Mn0.1Li	282.32 ± 5.11	219.81 ± 2.60	−0.3710	0.25	0.000045

**TABLE 4:** Fitting parameters in SBF.

Materials	Rs (Ωcm^2^)	Qdl (S.sec^n/m^2^)	n1 (0<*n*<1)	Rct (Ωcm^2^)	Cc (F/m^2^)	Rc (Ωcm^2^)	W (S.sec^5/cm^2^)
Zn	61.95	0.7875	0.74	577.6	24.37	30.66	0.02775
Zn0.8Mn	58.6	0.4185	0.6747	81.8	0.02064	1,607	0.01152
Zn0.8Mn0.1Li	64.22	0.1866	0.6226	17.88	0.005532	4200	1354000000
CaP Zn	85.77	0.006744	0.7084	815.7	0.006736	1,121	0.02532
CaP Zn0.8Mn	93.42	0.3512	0.6713	66.23	0.01724	1,257	0.02178
CaPZn0.8Mn.1Li	89.69	0.1385	0.5966	5,247	1.684	8595	0.00008695

**FIGURE 5:**
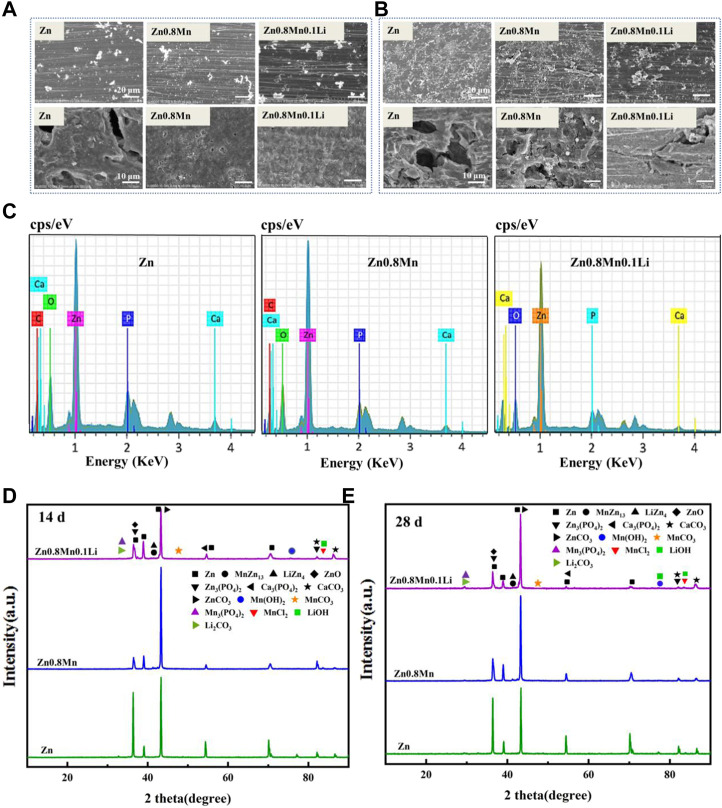
*In vitro* degradation tests. **(A)** Degradation morphology under FESEM after 14 days immersion in SBF. **(B)** Degradation morphology under FESEM after 28 days immersion in SBF. **(C)** The EDS spectra of Zn, Zn0.8Mn, and Zn0.8Mn0.1Li after 28 days immersion in SBF. **(D)** XRD diffraction patterns of degradation products after 14 days immersion in SBF. **(E)** XRD diffraction patterns of degradation products after 28 days immersion in SBF.

The samples were immersed in SBF solution for 14 and 28 days to observe the morphology of the corrosion products. After removing the corrosion products, the surface corrosion was also observed (Figure 5A). After 14 days of immersion, a small number of corrosion products were observed on the surface. The initial corrosion products on the surface of pure Zn and Zn-based alloys were round cloud aggregates. After removing the corrosion products, local corrosion pits appeared on the surface of pure Zn. While Zn0.8Mn and Zn0.8Mn0.1Li exhibited much more uniform degradation morphology. After soaking for 28 days, the corrosion products of the three alloys all increased to a certain extent. As shown in Figure 5B, the surface corrosion products of pure Zn and Zn0.8Mn showed an elongated crystal shape and accumulated in large quantities in local areas. After the removal of corrosion products, pure Zn and Zn0.8Mn showed characteristic uneven corrosion, accompanied by a large area of corrosion pits. However, the surface of Zn0.8Mn0.1Li showed flower-like corrosion products, which showed a smaller corrosion area and no obvious corrosion pits compared with the other two samples. All these results showed Zn0.8Mn0.1Li manifested more uniform degradation behavior. Additionally, the EDS analyses of the corrosion products after 28 days were performed. EDS spectrum showed that the white particles were mainly composed of Zn, P, O, C, and a small amount of Ca (Figure 5C). The degradation products were mainly ZnO, ZnPO_4_, CaPO_4_, ZnCO_3,_ and CaCO_3_ indicated from XRD patterns (Figures 5D,E). This result showed the diffraction peak of corrosion products was significantly enhanced after immersion for 28 days.

### 3.3 Surface Morphology and Composition of CaP coatings on Zn-based Alloys

The Zn/Fe coupling system includes zinc and iron clamp to form a point contact. Due to the corrosion potential difference, the iron clamp can be regarded as the coupled anode, zinc as the coupled cathode in the acid phosphate chemical conversion solution. And the initial power of this reaction was provided by the corrosion of the iron clamp. In addition, the transient high temperatures and high pressures generated by ultrasound on the Zn surface caused partial supersaturation of the ion concentration near the Zn surface, leading to heteronucleation growth of phosphate crystals. Finally, complete CaP coatings were formed.

**FIGURE 6:**
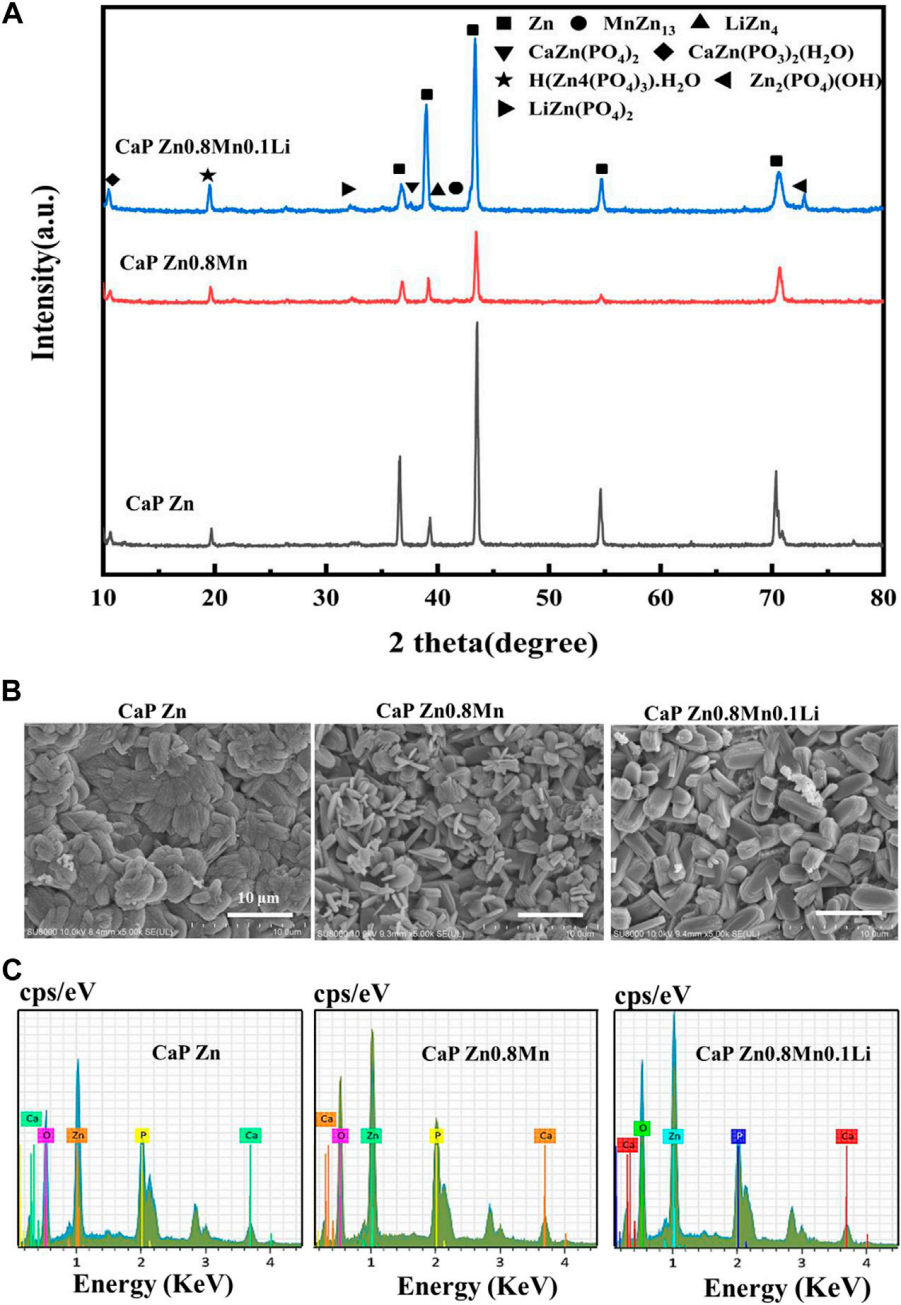
The microstructure and composition of CaP-coated Zn, Zn0.8Mn, and Zn0.8Mn0.1Li. **(A)** XRD spectrum. **(B)** Surface morphologies by FESEM. **(C)** The EDS spectra and relative contents of each element.

As can be seen from Figure 6A, CaP Zn, CaP Zn0.8Mn and CaP Zn0.8Mn0.1Li had similar XRD diffraction peaks. The phase composition were CaZn_2_(PO_4_)_2_, CaZn_2_(PO_3_)2(H_2_O), H(Zn_4_(PO_4_)_3_)H_2_O, and Zn_2_(PO_4_)(OH). The CaP Zn0.8Mn0.1Li consisted a small amount of LiZn(PO_4_)_2_ phase. The diffraction peak intensity of CaP Zn was obviously stronger than CaP Zn0.8Mn and CaP Zn0.8Mn0.1Li. The surface morphologies of CaP Zn, CaP Zn0.8Mn and CaP Zn0.8Mn0.1Li were shown in Figure 6B. Apparently, diverse dense and uniform coatings were formed on the surface of pure Zn and Zn-based alloys after chemical conversion. The morphologies between these three kinds of coatings were quite different. CaP Zn was composed of regular fibrous crystals that were tightly bound to each other without any gaps. Compared with CaP Zn, the microstructure of CaP Zn0.8Mn changed significantly and became relatively loose surface state composed of interwoven rod-like crystals. CaP Zn0.8Mn0.1Li was composed of uniform elliptic sheet crystals with regular shape and uniform sizes. As can be seen from Figure 6C, EDS results showed that the main elements of the coatings were O, P, Zn, and Ca, which confirmed the successful introduction of Ca and P elements on the coatings.

**FIGURE 7:**
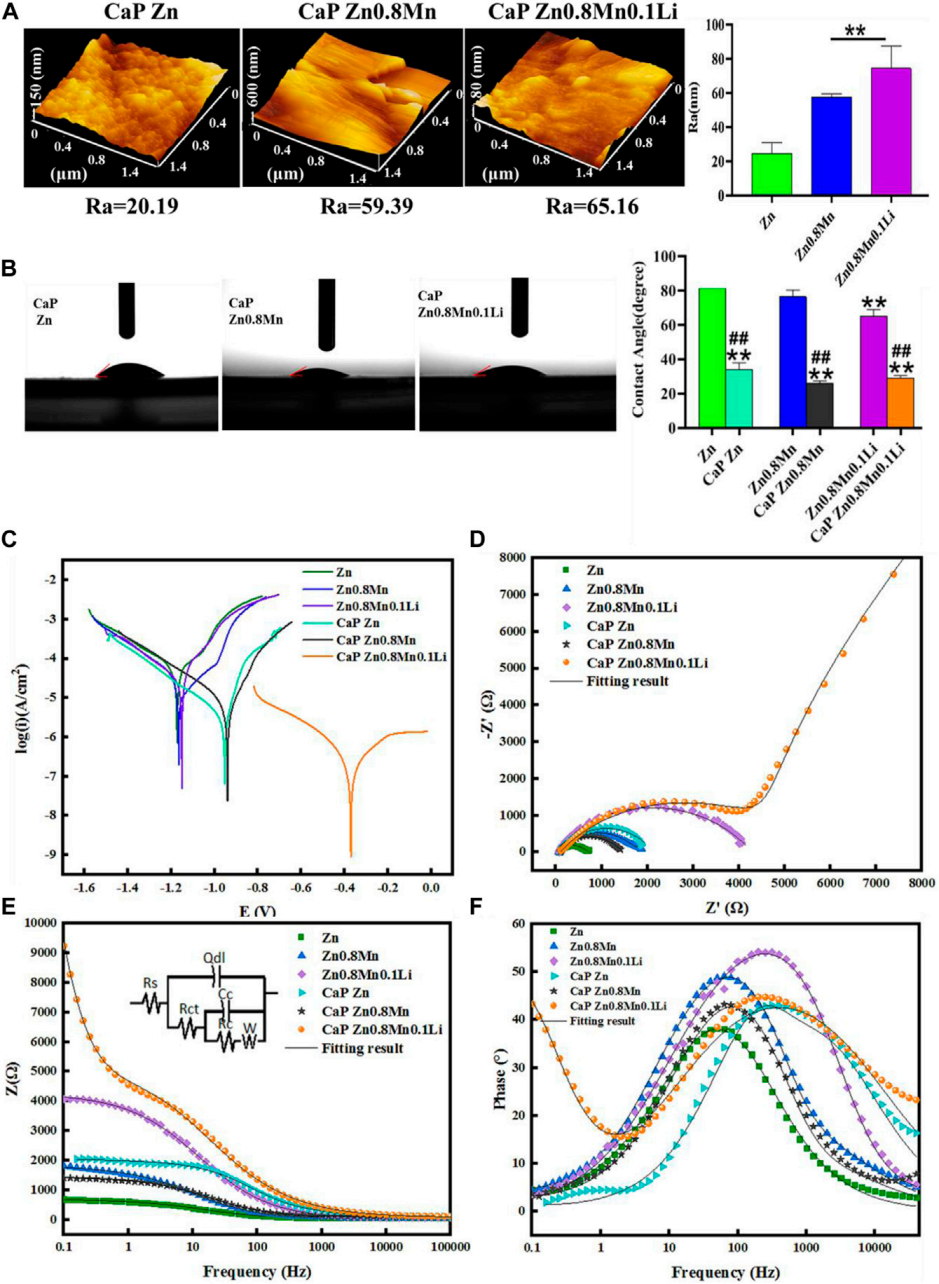
Characterization of CaP-coated Zn, Zn0.8Mn, and Zn0.8Mn0.1Li. **(A)** The surface roughness of the surfaces obtained by AFM. **(B)** Water contact angles. **(C)** Potentiodynamic polarization curves of CaP Zn, CaP Zn0.8Mn, and Zn0.8Mn0.1Li. **(D)** Nyquist plots. **(E)** Bode plots of |Z| vs Frequency. The insets in Figure 7E is equivalent circuit for fitting the EIS spectra. **(F)** Bode plots of phase angle vs Frequency. *p < 0.05 compared to pure Zn, **p < 0.01 compared to pure Zn, ##*p* < 0.01 compared to no coatings.

AFM test showed that the roughness of the samples surface increased significantly with the surface morphology (Figure 7A). Wettability results showed that both the CaP Zn, CaP Zn0.8Mn, and CaP Zn0.8Mn0.1Li became more hydrophilic (Figure 7B). The contact angle of CaP Zn, CaP Zn0.8Mn, and CaP Zn0.8Mn0.1Li decreased significantly, with values of 34.3 ± 3.66°, 29.9 ± 5.60°, and 29.0 ± 1.57°, respectively. However, there was a certain gap between the single particles of CaP Zn0.8Mn and CaP Zn0.8Mn0.1Li, resulting in a decrease in contact angle and an increase in hydrophilicity. Figure 7C showed the potentiodynamic polarization curve of the CaP coatings with pure Zn and Zn-based alloys. The electrochemical parameters calculated by Tafel extrapolation were shown in Table 2. Generally, the higher the corrosion voltage (Ecorr) and the lower the corrosion current density (Icorr) indicated that the corrosion rate was slower and the metal was more resistant to corrosion. As shown in Table 2, the Ecorr of CaP coatings were higher than the pure Zn and Zn-based alloys while the Icorr of CaP coatings were smaller, and the Icorr of CaP Zn0.8Mn0.1Li was the lowest, this showed the higher corrosion resistance of the CaP Zn0.8Mn0.1Li. As shown in the Nyquist curves (Figure 7D), CaP Zn0.8Mn0.1Li had a bigger semicircle diameter and had the largest impedance modulus (|z|) in the low-frequency region, which indicated its lower corrosion rate (Figure 7E). There were two-time constants in the Bode curve of CaP Zn0.8Mn0.1Li (Figure 7F), so two-time constant models with W diffusion impedance were used to fit the EIS data of all the specimens. The fitted equivalent circuit results were listed in Table 3, the Rc of CaP Zn0.8Mn0.1Li was the highest, so it had the highest corrosion resistance. These structural features were essential to enhance the osteogenesis-related cell attachment and accelerate tissue growth during osseointegration.

### 3.4 Biocompatibility of MC3T3-E1 cells

**FIGURE 8:**
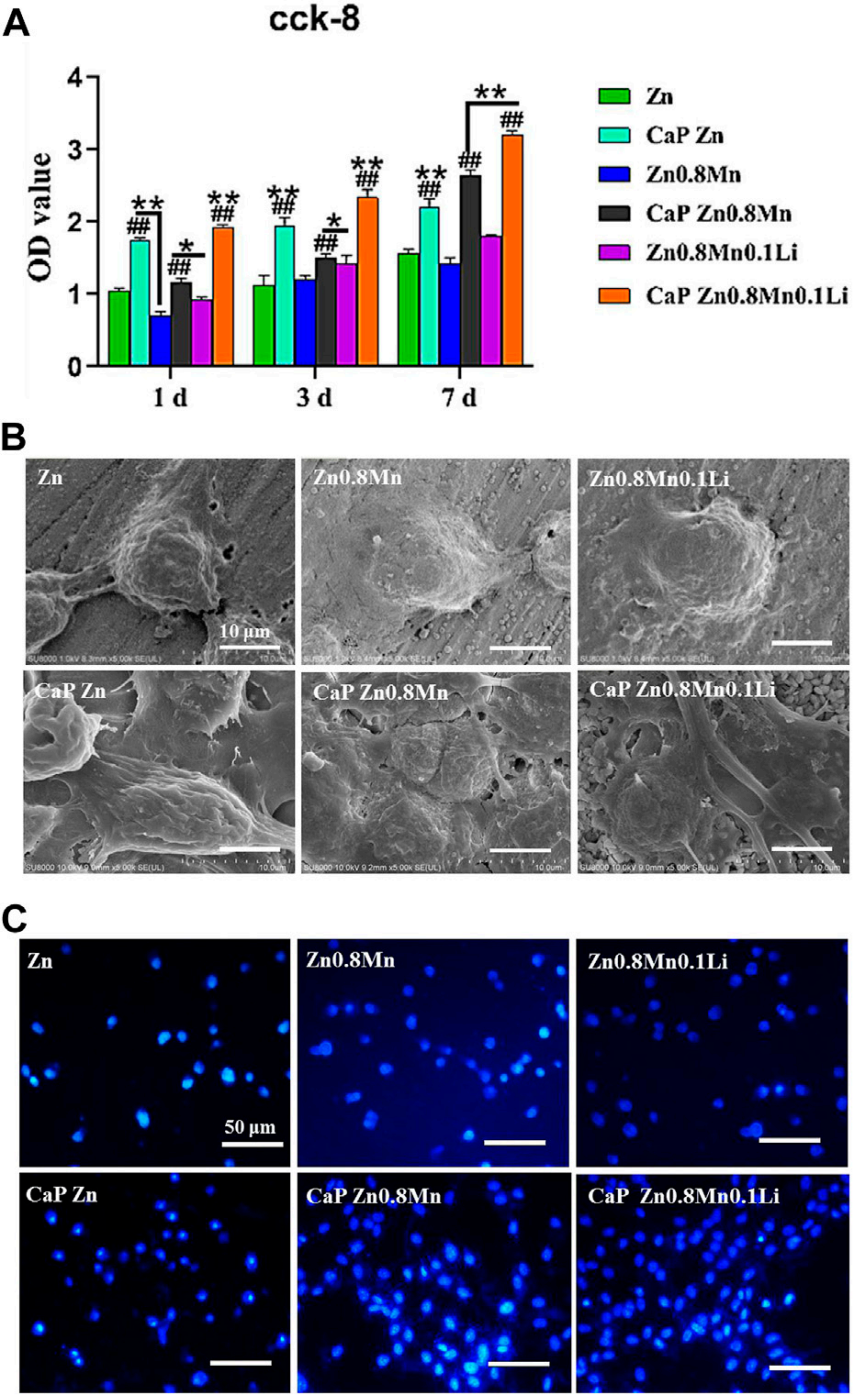
Cytocompatibility of Zn-based alloys and CaP-coated Zn-based alloys. **(A)** CCK-8 cells proliferation experiment of MC3T3-E1 after cultured 1, 3, and 7 days *in vitro*. **(B)** SEM and **(C)** DAPI nuclear staining pictures of MC3T3- E1 cells on different samples surface after cultured 3 days *p < 0.05 compared to pure Zn, **p < 0.01 compared to pure Zn, ##*p* < 0.01 compared to no coatings.

Good proliferation and adhesion of cells on scaffolds were two important factors in the construction of bone tissue engineering scaffolds. For the sake of estimating the proliferation ability of MC3T3-E1 cells cultured on scaffolds, CCK-8 was measured on days 1, 3, and 7 after cells inoculation. Results were shown in Figure 8A, the OD value of MC3T3-E1 cells was raised on day 3 and day 7. Compared with Zn and Zn0.8Mn, Zn0.8Mn0.1Li had a higher OD value and faster growth rate, which proved that Zn0.8Mn0.1Li had better biocompatibility. And Zn-based alloys were expected to become ideal bone osteogenesis materials. Moreover, compared with Zn, Zn0.8Mn, and Zn0.8Mn01Li, the OD value of the platforms coated with CaP coatings was significantly increased, and the CaP Zn0.8Mn0.1Li had the most considerable OD value, followed by CaP Zn0.8Mn and CaP Zn. The adhesive ability of MC3T3-E1 cells on the surfaces was evaluated by FE-SEM analysis and DAPI staining (Figures 8B,C). MC3T3-E1 cells were round on the surface of pure Zn and Zn-based alloys, with almost no pseudopods protruding. However, after adding CaP coatings, MC3T3-E1 cells were fusiform. It can be seen that MC3T3-E1 had obvious pseudopods protrusion on the surface of CaP Zn0.8Mn0.1Li, which connected with adjacent cells compactly. The number of attached cells increased significantly after Zn0.8Mn and Zn0.8Mn0.1Li were inoculated for 3 days while compared with the pure Zn. After adding CaP coatings, the number of MC3T3-E1 increased significantly, especially CaP Zn0.8Mn0.1Li.

**FIGURE 9:**
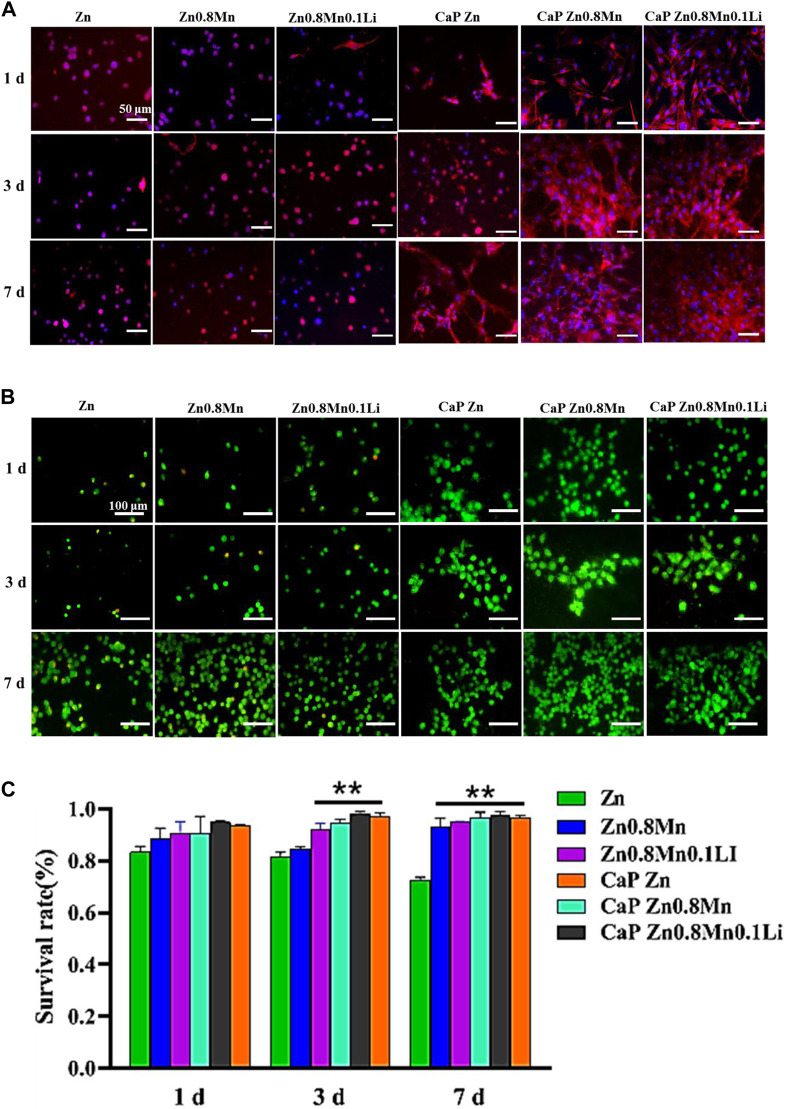
**(A)** The AM/PI staining results about cell activity *in vitro*. **(B)** Cell survival ratio by fluorescence expression quantity. **(C)** The morphology of MC3T3-E1 cells after seeding on the different scaffolds for 1, 3, and 7 days **p < 0.01 compared to pure Zn.

Figure 9A showed the staining of MC3T3-E1 cells on the scaffold surface. The red was the cytoskeleton factin and the blue was the nucleus. The cells cultured on the pristine Zn-based alloys showed a circular shape with very few pseudopods. The morphology of MC3T3-E1 on CaP Zn was slightly expanded on day 7. And the morphology of MC3T3-E1 revealed spindle-like shapes on the CaP Zn0.8Mn and CaP Zn0.8Mn0.1Li. Compared with MC3T3-E1 on the CaP Zn0.8Mn, the sizes of the MC3T3-E1 were wider on the CaP Zn0.8Mn0.1Li, and it was fully paved on 7 days. AM/PI staining was performed to assess cell viability and calculate survival rate. As shown in Figure 9B, when cells were dyed with AM/PI, dead cells showed typical red fluorescence, while living cells showed green fluorescence. The survival rate of the pure Zn group showed a downward trend, and a large number of dead cells occurred on day 7. These results showed that the cells had a higher survival rate at Zn0.8Mn and Zn0.8Mn0.1Li than pure Zn. (Figure 9C). No dead cells were found in the coating group, therefore, the biocompatibility of CaP coatings was good. The results showed very little or no effect on the survival rate of MC3T3-E1 cells compared to no coated. Confocal laser scanning microscope (CLSM) analysis was used to observe the morphology of cell attachment and spreading after being cultured for 1, 3, and 7 days.

**FIGURE 10:**
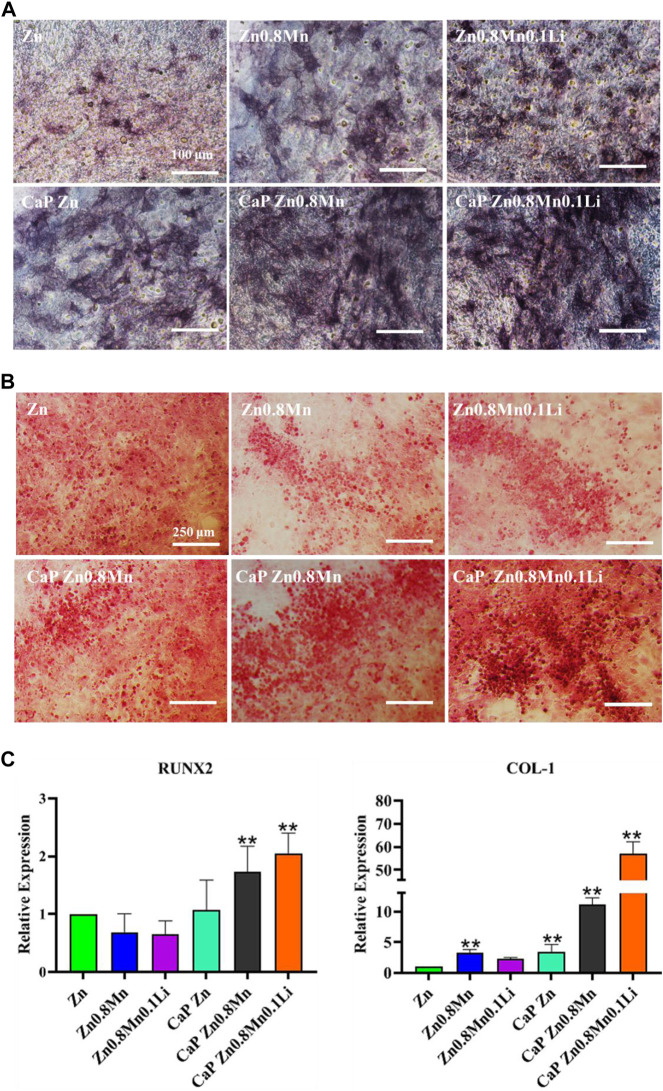
Osteogenic differentiation of MC3T3-E1 cells. **(A)** ALP staining and quantification in MC3T3-E1 cells. **(B)** Alizarin red S staining and quantification in MC3T3-E1 cells. **(C)** The expression of osteogenic gene was detected by qRT-PCR. *p < 0.05, **p < 0.01 represent significant differences between the indicated columns.

For the sake of the early stage osteogenic activity of scaffolds, we observed the ALP staining of MC3T3-E1 cells cultured on different scaffolds for 7 days. As shown in Figure 10A, only a small amount of gray deposits can be observed on the pure Zn, the gray deposits of Zn0.8Mn0.1Li was obviously more and darker than that of pure Zn. After the introduction of CaP coatings on the Zn-based alloys, the gray sediments had increased to a certain extent. However, more black sediments were produced on CaP Zn0.8Mn0.1Li. Calcium deposition reflected the efficiency of the mineralization stage. After MC3T3-E1 cells were inoculated on different scaffolds for 14 days, the mineralization of calcium nodules was measured by ARS. According to Figure 10B, a small raise in the number of calcium nodules was surveyed on Zn0.8Mn and Zn0.8Mn0.1Li alloys compared with that of pure Zn, but there were quite a few calcium nodules on the CaP-coated Zn-based alloys. This showed that surface-modified CaP was more conducive to the secretion of mineralized extracellular matrix and can promote osteogenic activity, especially CaP Zn0.8Mn0.1Li. The expression of osteogenic genes COL1 and RUNX2 were detected by RT-PCR. From RT-PCR results, we can see the expression of osteogenic genes in MC3T3-E1 on CaP-coated Zn-based alloys was significantly higher than pure Zn. Especially the CaP Zn0.8Mn0.1Li had the highest expression of osteogenic genes (Figure 10C). These results indicated that CaP Zn0.8Mn0.1Li can effectively promote the osteogenic differentiation of MC3T3-E1 cells.

## 4 Discussion

In this study, we first self-designed high-strength Zn0.8Mn0.1Li alloy to study the performance of Zn-based alloys in orthopedic implants. According to Zn-Mn phase diagram, the solid solubility of Mn in Zn reached a maximum of 0.8% at eutectic reaction temperature (Shi et al., 2018). With the addition of alloying elements, the microhardness of the alloy is significantly improved, which can meet the requirements of clinical orthopedic implants. This was attributed to that MnZn_13_ particles stimulated the recrystallization of Zn grains at room temperature and released the internal stress during the tensile test (Sun et al., 2021). Further alloying 0.1% Li in Zn-Mn alloy introduced finer LiZn_4_ second phase, refined the grain, and significantly improved its strength. The formation of the second phase can also avoid serious pitting and local corrosion in the degradation process of Zn alloys.

**FIGURE 11:**
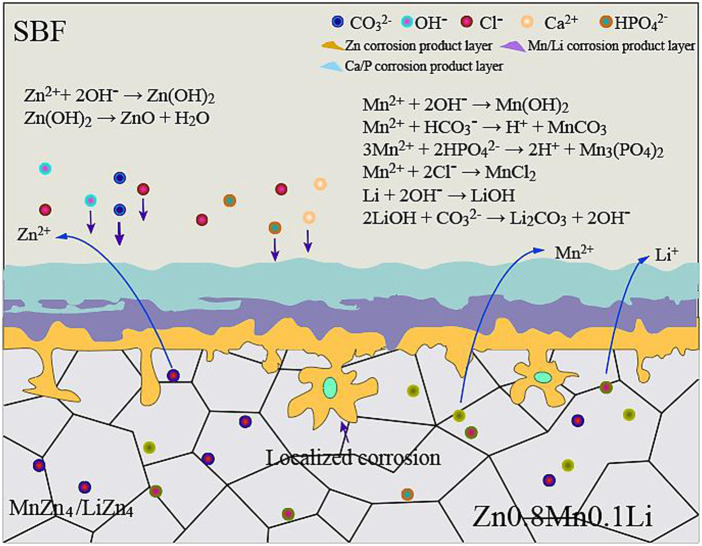
Corrosion mechanism diagram of Zn0.8Mn0.1Li alloy in SBF.

It is positive impact on the corrosion resistance of Zn-Mn alloy for the addition of Li because the structure of Zn0.8Mn0.1Li alloy was more uniform and finer. When Zn0.8Mn0.1Li alloy was immersed in SBF, Zn controlled the anodic and cathodic reactions, which accounted for more than 98 wt% of the alloy (Li et al., 2020; Sun et al., 2021). The corrosion mechanism diagram of Zn0.8Mn0.1Li alloy in SBF was shown in Figure 11. The reactions were as follows:

$$Zn\to {Zn}^{2+}+e^{-}$$ (1)

$$O_{2}+2H_{2}O+4e^{-}\to 4OH^{-}$$ (2)

$${Zn}^{2+}+2{OH}^{-}\to Zn{\left(OH\right)}_{2}$$ (3)

The dissolved Zn^2+^ can severally draw HPO_4_^2-^ and Ca^2+^ in the SBF, resulting in the deposition of Ca_3_(PO_4_)_2_, CaCO_3_, Zn_3_(PO_4_)_2_, and ZnCO_3_, which were specific corrosion products of Zn and its alloys in physiological environment (Ma et al., 2016; Yang et al., 2016; Zhao et al., 2019). Partial Zn(OH)_2_ was seemly to be dehydrated to produce ZnO through subsequent reaction:

$${Zn}^{2+}+{HPO}_{4}^{2-}/{CO}_{3}^{2-}\to {Zn}_{3}{\left({PO}_{4}\right)}_{2}/{ZnCO}_{3}$$ (4)

$${Ca}^{2+}+{HPO}_{4}^{2-}/{CO}_{3}^{2-}\to {Ca}_{3}{\left({PO}_{4}\right)}_{2}/{CaCO}_{3}$$ (5)

$$Zn{\left(OH\right)}_{2}\to ZnO+H_{2}O$$ (6)

The ions released from the alloy were primarily Zn^2+^, Mn^2+,^ and Li^+^. Guided by the XRD profile in Figures 5D,E, the metallic ions (Mn^2+^ and Li^+^) can respectively attract with OH^−^, HCO_3_^−^, HPO_4_^2-^, and Cl^−^ in the SBF as follows:

$${Mn}^{2+}+2Ο{Η}^{-}\to Mn{\left(OH\right)}_{2}$$ (7)

$${Mn}^{2+}+Η{CO}_{3}^{-}\to H^{+}+{MnCO}_{3}$$ (8)

$$3{Mn}^{2+}+2Η{PO}_{4}^{2-}\to 2H^{+}+{Mn}_{3}{\left({PO}_{4}\right)}_{2}$$ (9)

$${Mn}^{2+}+2Cl^{-}\to Μ{nCl}_{2}$$ (10)

Li showed great fluidity and activity in the corrosive layer and was easy to react with dissolved O_2_ in the water environment, forming Li_2_O in the outer corrosive layer (Li et al., 2020). Combined with XRD (Figure 1), water-insoluble lithium-rich corrosion products were formed according to the following reactions:

$$2Li+2H_{2}O\to 2LiOH+{Η}_{2}$$ (11)

$$2LiOH+{CO}_{3}^{2-}\to {Li}_{2}{CO}_{3}+2{OH}^{-}$$ (12)

The results of *in vitro* experiments showed that all samples had no toxic side effects on cell proliferation and adhesion, showing a good proliferation trend. But experiments had demonstrated that Zn-based alloys had certain effects on the morphology of MC3T3-E1 cells, the cells do not spread well on it. However, the spread area of osteoblasts on CaP Zn0.8Mn0.1Li was larger, and the aspect ratio was about 2:1. Relevant studies had also confirmed that the cytoskeleton guides cells to regulate their adhesion or other behaviors to adapt to the corresponding microenvironment (Jing et al., 2021). It had been reported that more cell contact and larger cell spread are conducive to osteogenic differentiation of MC3T3-E1 cells (Yao et al., 2013c). This may be the reason for the highest expression of osteogenic characteristic genes under CaP Zn0.8Mn0.1Li stimulation (Yao et al., 2019). A similar elongated cell morphology had also been reported to increase cell viability, which may provide another advantage for CaP Zn0.8Mn0.1Li scaffolds in improving cell viability and cell proliferation (Zou et al., 2021).

The survival rate of Zn0.8Mn and Zn0.8Mn0.1Li alloys showed an increasing trend, which indicated that the processed Zn-based alloys had certain biocompatibility. However, the cell survival rate on the surface of pure Zn showed a downward trend, which may be caused by the rapid degradation of pure Zn and the high local Zn^2+^ concentration. It has been reported that the cytocompatibility of pure Zn and Zn alloys exhibited a concentration-dependent phenomenon, the low concentrations of Zn^+^ were beneficial to cells and high concentrations were harmful to cells. For example, Ma demonstrated that Zn^2+^ promoted cell proliferation at low concentrations of 60 μM and inhibit cell proliferation at concentrations greater than 80 μM (Ma et al., 2015).

The additions of Mn and Li to Zn-based alloys have statistically modified the biocompatibility to some extent. Mn is the immune system and a variety of enzymes necessary trace elements play an important role in bone growth and development of animals. Mn deficiency can lead to poor bone growth, reproductive disorders, and neurological disorders (Sun et al., 2021). Li is also necessary for human body nutrition elements, the latest research showed that Li can stimulate the osteogenic differentiation of bone marrow mesenchymal stem cells and promote bone reconstruction (Li et al., 2020).

It was considered that the coexistence of Mn^2+^ and Li + promoted Mn^2+^ and Li^+^ influx into MC3T3-E1 cells, inhibited Zn^2+^ effusion, effectively activated Runx2, and induced osteogenic differentiation of MC3T3-E1 cells (Yu et al., 2017). The osteogenic effect of Zn0.8Mn0.1Li may be attributed to the synergistic effect of Li^+^ and Zn^2+^. Although the content of Li (0.1%) is low in the alloy, it is preferentially released during degradation due to its high chemical reactivity. This can effectively regulate the release of Zn^2+^ and avoid the adverse effect of excessive local zinc ion concentration on the osteogenic effect.

In the future design of Zn-based alloys for orthopedic, we should select elements with osteogenic effects and low electrode potential to balance their synergistic effects and reduce the release rate of Zn^2+^ moderately. Therefore, Zn0.8Mn0.1Li alloy had good comprehensive properties and was expected to become a candidate material for the development of biodegradable alloys.

The physicochemical properties of the material surface were the key factors to regulate the bone-implant bonding. The adhesion, proliferation, and differentiation of cells were intensely dependent on the surface of materials, including surface composition, charge, and hydrophilicity. Such as the effect of roughness on protein adsorption will directly affect cell adhesion and diffusion (Yang et al., 2021; Yuan et al., 2022). It has been shown that cell fate can be determined by designing the surface topography to give specific physicochemical parameters (Priyadarshini et al., 2019). Phosphate chemical conversion technology is an effective technique to adjust the surface morphology and element composition by forming different transformation films on the surface of implants. The formation of phosphate can effectively slow down the corrosion rate, mainly because of the low solubility product constant of phosphate. Calcium phosphate was the main component of hard tissue, so it had a positive effect on the adhesion and growth of bone cells on biomaterials (Su et al., 2019). Moreover, the hydrophilicity and roughness of the scaffolds were significantly improved after the preparation of CaP coatings on the scaffolds, which were more suitable for cell attachment (Ling et al., 2021). It is necessary to accelerate bone repair in the process of promoting bone regeneration. Zn0.8Mn0.1Li alloy will be a promising bone implant to achieve these results. The study proved CaP coatings could significantly promote bone regeneration.

## 5 Conclusion

All in all, to satisfy the clinical demands of osteogenic ability, biocompatibility, and biodegradability of medical degradable metal materials, a high-strength Zn0.8Mn0.1Li alloy was designed. To further improve its cytocompatibility, we modified its surface and prepared calcium-phosphorus coatings. It decreased the ion release and subsequently also decreased toxicity and enhanced MC3T3-E1 cells proliferation and adhesion. Therefore, this study will propose an innovative strategy for the surface design of biomedical implants.

## Data Availability

The raw data supporting the conclusion of this article will be made available by the authors, without undue reservation.
